# Insights into Medication-Induced Osteonecrosis of the Jaw Through the Application of Salivary Proteomics and Bioinformatics

**DOI:** 10.3390/ijms252212405

**Published:** 2024-11-19

**Authors:** Vladimíra Schwartzová, Galina Laputková, Ivan Talian, Miroslav Marcin, Zuzana Schwartzová, Dominik Glaba

**Affiliations:** 1 Clinic of Stomatology and Maxillofacial Surgery, Faculty of Medicine, University of P. J. Šafárik and Louis Pasteur University Hospital, 041 90 Košice, Slovakia; vladimira.schwartzova@upjs.sk (V.S.); zuzana.schwartzova@upjs.sk (Z.S.); 2 Department of Medical and Clinical Biophysics, Faculty of Medicine, University of P. J. Šafárik, 040 11 Košice, Slovakia; ivan.talian@upjs.sk (I.T.); miroslav.marcin@upjs.sk (M.M.); 3 Faculty of Medicine, University of P. J. Šafárik, 041 90 Košice, Slovakia; dominik.glaba@student.upjs.sk

**Keywords:** medication-related osteonecrosis of the jaw, proteomics, bioinformatic analysis, saliva

## Abstract

Long-term treatment with bisphosphonates is accompanied by an increased risk of medication-related osteonecrosis of the jaw (MRONJ). Currently, no clinically useful biomarkers for the predictive diagnosis of MRONJ are available. To investigate the potential key proteins involved in the pathogenesis of MRONJ, a proteomic LC-MS/MS analysis of saliva was performed. Differentially expressed proteins (DEPs) were analyzed using BiNGO, ClueGO, cytoHubba, MCODE, KEGG, and ReactomeFI software packages using Cytoscape platforms. In total, 1545 DEPs were identified, including 43 up- and 11 down-regulated with a 1.5-fold cut-off value and *adj*. *p*-value < 0.05. The analysis provided a panel of hub genes, including *APOA2*, *APOB*, *APOC2*, *APOC3*, *APOE*, *APOM*, *C4B*, *C4BPA*, *C9*, *FGG*, *GC*, *HP*, *HRG*, *LPA*, *SAA2-SAA4*, and *SERPIND1*. The most prevalent terms in GO of the biological process were macromolecular complex remodeling, protein–lipid complex remodeling, and plasma lipoprotein particle remodeling. DEPs were mainly involved in signaling pathways associated with lipoproteins, the innate immune system, complement, and coagulation cascades. The current investigation advanced our knowledge of the molecular mechanisms underlying MRONJ. In particular, the research identified the principal salivary proteins that are implicated in the onset and progression of this condition.

## 1. Introduction

Medication-related osteonecrosis of the jaw (MRONJ) represents a rare but nonetheless serious adverse drug reaction. It is associated with necrosis of the maxillofacial bones [1]. MRONJ encompasses bisphosphonate-related osteonecrosis of the jaw [2], denosumab-related osteonecrosis of the jaw [3], and angiogenesis inhibitor-induced osteonecrosis of the jaw [4].

Bisphosphonates are a class of calcium-binding drugs used to prevent bone resorption in the treatment of malignant bone metastases, multiple myeloma, metabolic bone diseases, including osteoporosis, and Paget’s disease. Long-term use of bisphosphonates is correlated with an increased risk of osteonecrosis. The pathological mechanisms underlying the development of MRONJ are influenced by a multitude of factors that collectively suppress bone resorption through the induction of osteoclast apoptosis [5] and exert direct toxic effects on the oral epithelium [6]. Bisphosphonates have been demonstrated to regulate the activity of various cells with a role in immune system dysfunction [7]. These agents are also associated with oral infection and inflammation [8], inhibition of angiogenesis [9], and systemic comorbidities [7].

It is notable that the jawbone appears to be the most susceptible to damage in cases of osteonecrosis induced by bisphosphonates, particularly in comparison to other bones and joints affected by this condition. It has been suggested that this phenomenon may be the result of an accelerated rate of bone turnover in the maxilla. An alternative hypothesis posits that bisphosphonates do not merely affect bone but also impact multiple elements of adjacent tissue, including fibroblasts and vascular structures [10].

Currently, there is no established, clinically useful biomarker for the predictive diagnosis of jaw osteonecrosis. Despite the multitude of studies conducted thus far, no one has been able to conclusively identify a single protein, gene, or metabolite as the sole driver of MRONJ development.

Previous studies have identified a potential correlation between the onset of MRONJ and the activity of specific genes. The authors examined gene expression data of individuals with multiple myeloma obtained from microarray experiments and discovered seven hub genes (*ACTB*, *FN1*, *GAPDH*, *JUN*, *PTPRC*, *STAT3*, and *TNF*) associated with bisphosphonate-induced osteonecrosis of the jaw [11]. *GAPDH* is among the most significant genes and a pivotal enzyme in glycolysis. Beyond this, *GAPDH* is implicated in multiple cancer-related biological processes, having recently emerged as a primary focus in cancer research. It has been reported that *GAPDH* is frequently dysregulated in numerous cancer types. Consequently, the alteration of *GAPDH* expression in MRONJ in patients with multiple myeloma should be approached with caution.

Integrative analyses yielded the prioritization of three salivary proteins (*SERPINA3*, *HBD*, and *MMP9*) [12] and 17 hub genes and hsa-mir-16-1, hsa-mir-21, hsa-mir-23a, hsa-mir-145, hsa-mir-186, hsa-mir-221, and hsa-mir-424 [13] associated with MRONJ.

Similarly, the authors of study [14] selected *MMP9* and *DSP* from the 200 differentially expressed proteins (DEPs) identified in patients with bisphosphonate-related osteonecrosis of the jaw for subsequent validation. The majority of these proteins were postulated to be involved in drug metabolism and in immunological and dermatological diseases.

Increased production of IL-10, IL-28, OPG, and osteocalcin was seen in the postoperative exudate from oral lesions after bisphosphonate medication. As opposed to the control, there was a reduction in the expression of TNFα and LL-37 for MRONJ [15]. Additionally, the study indicated that *KRT18* and *PABPC3* may serve as crucial molecular markers for the disease [16].

The dominant hypothesis among scientific studies is that MRONJ is predominantly linked to signaling pathways involving immunoglobulin, cell adhesion, and cytoskeleton [17]. Further findings from a proteomic examination [18] demonstrated that treatment with bisphosphonates substantially modifies the RIPK3/Wnt/GSK3/β-catenin signaling pathway resulting in aberrant remodeling, osteogenesis, anabolism, and angiogenesis [15]. Nevertheless, systematic and bioinformatic study has also indicated the potential for disruptions of signaling pathways in interleukin signaling and in maintaining homeostasis [13].

The potential for early-stage necrosis of the jaw to be detected by biomarkers in body fluids is an opportunity for more effective and curative management. In the context of oral cavity disorders, saliva represents the biofluid most proximate to oral mucosal lesions. Saliva, through repeated analysis, allows for real-time monitoring of the treatment response and resistance of MRONJ. In addition, liquid biopsy is a relatively low-risk, non-invasive procedure [19].

Moreover, the application of modern mass spectrometry techniques to the proteomic analysis of saliva has facilitated the rapid evaluation of numerous candidate biomarkers and the identification of specific signatures in association with various forms of oral pathologies. This approach has allowed for the examination of a large range of specimens and has given important new information on the potential uses of saliva as a diagnostic tool.

In this study, we used high-throughput label-free mass spectrometry and bioinformatics approaches to investigate the salivary proteomes of healthy subjects and patients with MRONJ. Our goal was to identify differentially expressed proteins potentially involved in bisphosphonate-induced osteonecrosis of the jaw and to propose multi-protein panels with the potential to discriminate individuals with MRONJ. Understanding the salivary proteome has important clinical implications. It aids in the identification of potential biomarkers for early diagnosis and risk stratification in MRONJ. It also opens the possibility of reducing the adverse effects of bisphosphonates in MRONJ through targeted therapies aimed at modulating specific protein regulatory patterns. From basic research to clinical application, these findings have broad potential implications for the design of future studies.

## 2. Results

**Figure 1:**
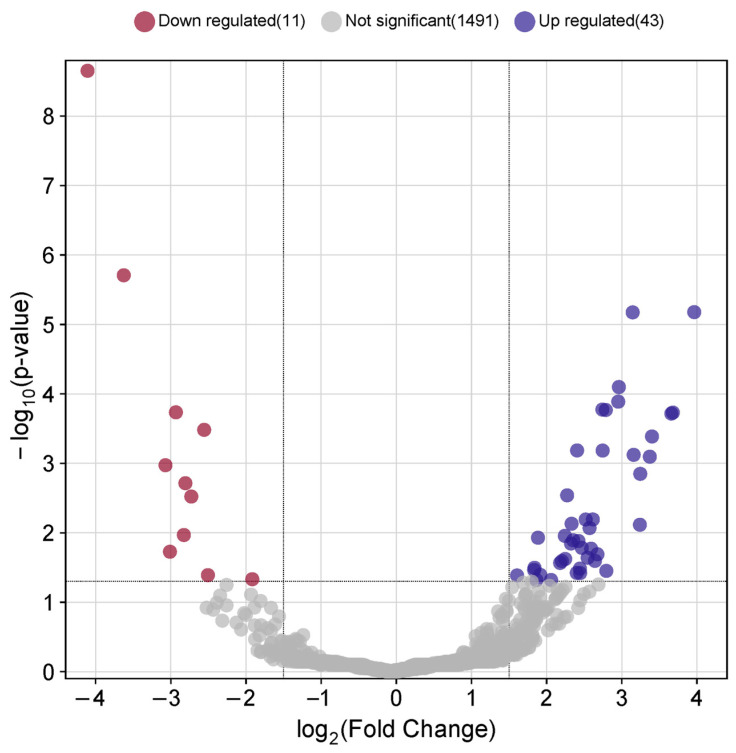
Volcano plot of differentially expressed proteins in MRONJ. In the saliva of MRONJ patients, there were 11 significant down-regulated DEPs and 43 significant up-regulated DEPs.

Salivary proteomic data were analyzed to identify signatures of MRONJ, focusing on salivary proteins that showed significant fold changes compared to those of healthy individuals. The screening threshold for identifying DEPs between the MRONJ and control groups was set at a 1.5-fold cutoff value and an *adj. p* < 0.05. Using a proteomic approach, 1545 DEPs (Table S1) in total were found in saliva. As shown in Figure 1, a set of 54 DEPs were identified under these conditions, including 11 down-regulated and 43 up-regulated DEPs. The top ten differentially expressed proteins in MRONJ that are considerably up- and down-regulated as determined by fold change are included in Table 1.

### 2.1. Protein–Protein Network Analyses

**Figure 2:**
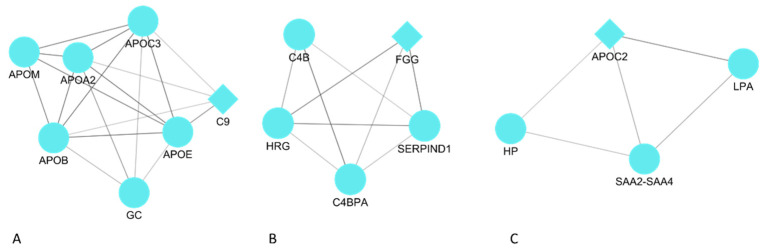
The MCODE analysis of the top three clusters. A diamond-shaped node highlights the seed protein involved in cluster formation. (**A**) cluster I; (**B**) cluster II; (**C**) cluster III.

To determine the interrelationships among DEPs associated with MRONJ, a gene interaction network analysis was performed. A network imported from the STRING database to Cytoscape was used to illustrate the interactions within the prognosis-related network. A score of >0.4 was applied as the threshold for determining the relevant data set. The MCODE clustering algorithm was employed to extract highly interconnected clusters from the primary protein interaction network, which had been constructed previously. Figure 2 shows three clusters derived using the MCODE method.

**Figure 3:**
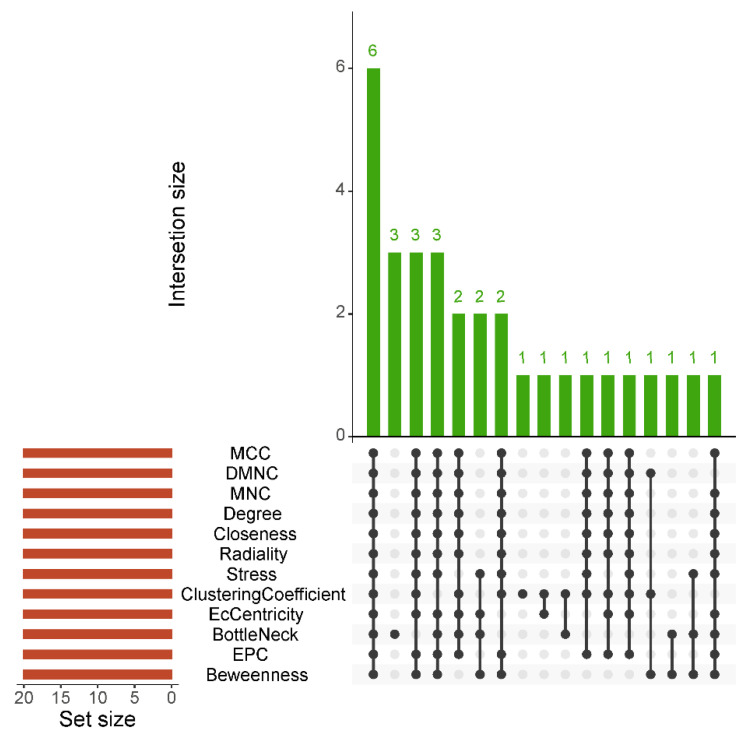
The UpSet plot of DEPs associated with MRONJ. The plot represents the intersection between the 12 types of local-based and global-based CytoHubba methods applied to the analysis of the top 20 highly interconnected hub DEPs.

**Figure 4:**
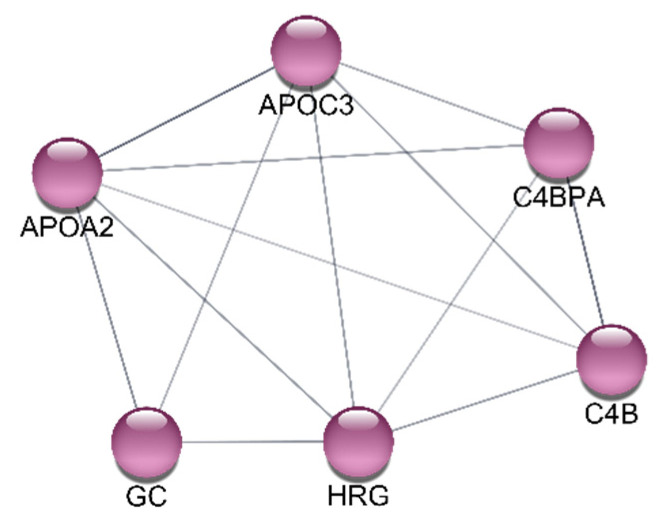
Gene interaction network plot of hub DEPs associated with MRONJ. In the gene interaction network generated using Cytoscape, nodes represent proteins/genes, and lines indicate interactions.

Network topology analysis was performed using the CytoHubba plugin for Cytoscape. For each of the twelve algorithms of CytoHubba, the top 20 most highly connected hub genes were identified within the entire complex network of DEPs. Consequently, six genes were identified at the intersection of all methods used: *APOA2*, *APOC3*, *C4B*, *C4BPA*, *GC*, and *HRG* (Figure 3 and Figure 4). The results demonstrated that all the hub genes exhibited intrinsic gene–gene interactions within the STRING network, thereby suggesting their potential critical impact on the prognosis of MRONJ incidence.

A set of genes obtained by the union of MCODE and CytoHubba analyses were subjected to further analysis. This group of genes was composed of three MCODE clusters and a group of six highly interconnected hub genes: *APOA2*, *APOB*, *APOC2*, *APOC3*, *APOE*, *APOM*, *C4B*, *C4BPA*, *C9*, *FGG*, *GC*, *HP*, *HRG*, *LPA*, *SAA2-SAA4*, and *SERPIND1*.

### 2.2. Functional Enrichment Analysis

**Figure 5:**
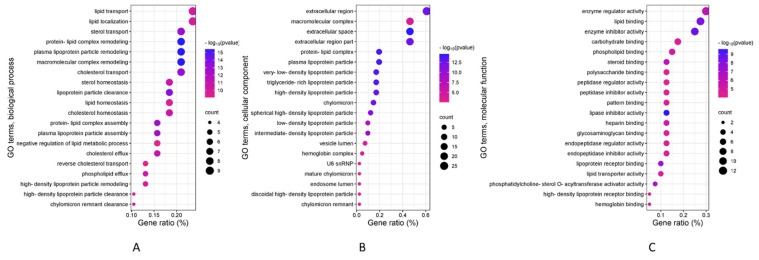
Gene ontology terms for MRONJ-associated genes. The Benjamini and Hochberg multiple testing procedure was employed at the cut-off criterion of *p* < 0.05. (**A**) the top 20 significant GO BP terms; (**B**) the top 20 significant GO CC terms; (**C**) the top 20 significant GO MF terms.

Afterwards, to assess the potential influence of DEPs on the biological functions of corresponding genes in MRONJ, GO enrichment analyses were conducted. The results showed that in total, 318 significant terms within the GO BP categories had a strong correlation with MRONJ, encompassing macromolecular complex remodeling, protein–lipid complex remodeling, and plasma lipoprotein particle remodeling (Figure 5A). Moreover, 21 markedly disparate terms within the GO CC category, including those pertaining to plasma lipoprotein particles and protein–lipid complexes, were identified (Figure 5B). Additionally, 60 terms in the GO MF category (Figure 5C) were identified. The most significantly enriched functions for the MF were those associated with genes involved in lipid binding, lipase inhibitor activity, and lipoprotein receptor binding.

**Figure 6:**
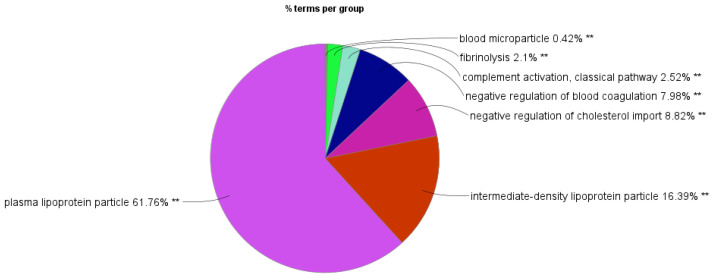
Functionally related groups of enriched GO terms in differentially expressed salivary proteins of MRONJ patients. The enrichment of GO terms was accomplished using the ClueGo application implemented in Cytoscape. Functionally related pathway terms are clustered according to color; ** *p* < 0.01.

In addition, a set of genes obtained from MCODE and CytoHubba analyses was subjected to GO functional analysis for comparison (Figure S1; Figure 6). This group included proteins/genes from three MCODE clusters and a group of six highly related hub genes: *APOA2*, *APOB*, *APOC2*, *APOC3*, *APOE*, *APOM*, *C4B*, *C4BPA*, *C9*, *FGG*, *GC*, *HP*, *HRG*, *LPA*, *SAA2-SAA4*, and *SERPIND1*.

While several clusters can be distinguished in the set of 122 BP GO terms, such as complement activation, classical pathway, negative regulation of cholesterol import, negative regulation of blood coagulation, and fibrinolysis, processes linked to genes that are implicated in high-density lipoprotein particle clearance and remodeling of the protein–lipid complex were most significantly enriched. Moreover, whereas the GO MF terms of DEPs were highly enriched for processes involved in lipoprotein particle receptor binding, heparin binding, lipase inhibitor activity, and high-density lipoprotein particle receptor binding, lipoprotein particle-related terms showed significant enrichment for the CC category.

### 2.3. Enrichment and Pathway Analysis

**Figure 7:**
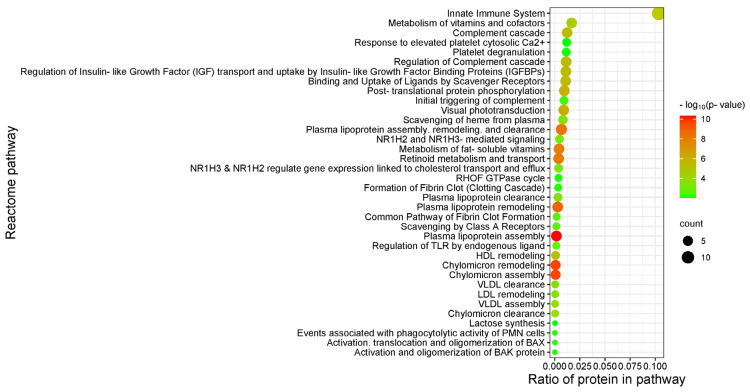
Enriched ReactomeFI pathways in differentially expressed salivary proteins of MRONJ patients. Only significantly enriched networks (*p*-value < 0.05; Benjamini and Hochberg) are displayed.

In order to ascertain the functional relevance of the genes associated with MRONJ, a ReactomeFI pathway analysis was conducted using the Cytoscape platform. The analysis showed predominant enrichment of DEPs in pathways related to plasma lipoprotein assembly, chylomicron remodeling, and chylomicron assembly (Figure 7).

**Figure 8:**
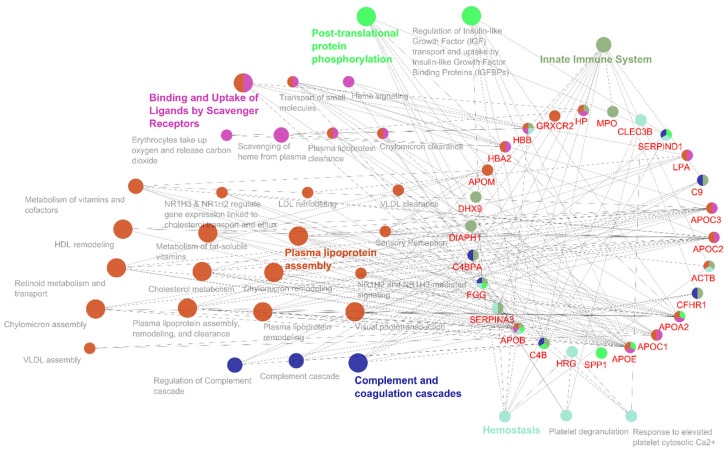
Color-coded presentation of enriched KEGG and Reactome pathways of functionally related groups. The enrichment of pathways was accomplished using the ClueGo application implemented into Cytoscape. The label of the most significant term within each group is highlighted. The size of a node is directly proportional to the number of protein entities contained within it. Only those networks exhibiting a significant degree of enrichment are displayed (*p*-value < 0.05; Benjamini and Hochberg).

**Figure 9:**
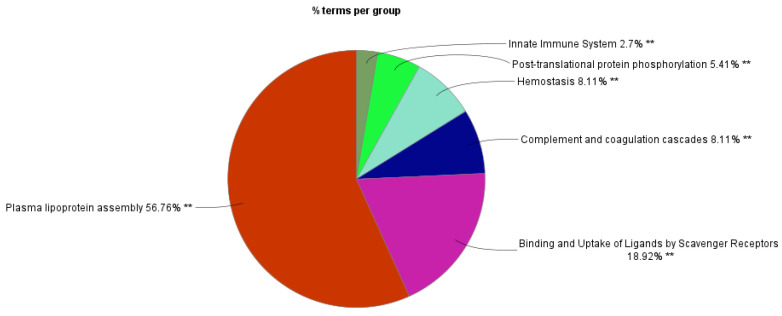
Functionally related groups of enriched pathways in differentially expressed salivary proteins of MRONJ patients. The enrichment of KEGG and Reactome pathways was accomplished using the ClueGo application implemented into Cytoscape. Functionally related pathway terms are clustered according to color; ** *p* < 0.01.

Furthermore, we conducted a network analysis of the MRONJ-specific DEPs using the ClueGO extension in Cytoscape (Figure 8 and Figure 9). The evaluation was conducted using the Reactome and KEGG signaling pathways databases, with the results subsequently filtered using the Benjamini and Hochberg method to identify pathways with a *p*-value less than 0.05. A network examination revealed six parent pathways, among which were plasma lipoprotein assembly, R-HSA:8963898; post-translational protein phosphorylation, R-HSA:8957275; innate immune system, R-HSA:168249; complement and coagulation cascades, KEGG:04610; binding and uptake of ligands by scavenger receptors, R-HSA:2173782; and hemostasis, R-HSA:109582. Genes from all clusters were associated with 65 representative terms and pathways with Final KappaScore groups = 6.

## 3. Discussion

Reliable biomarkers for MRONJ prognosis and prediction continue to be a major difficulty in the area, despite substantial research. Many studies on MRONJ have employed serum and cell cultures as the experimental medium [18,20]. Saliva proteomics, coupled with a bioinformatics analysis integrating PPI network analysis, GO, and signaling pathway enrichment strategies, may serve as an additional methodological approach to complement existing research on putative biomarkers of MRONJ.

The exact molecular processes that underlie MRONJ are still unknown, and the way in which it manifests itself in relation to salivary protein expression in the oral cavity is also not entirely understood. However, our findings indicate that human saliva contains proteomic biomarkers with the potential to differentiate between individuals with and without MRONJ.

A total of 1545 DEPs were identified in saliva based on a proteomic strategy that may offer new perspectives on the underlying mechanisms of MRONJ pathogenesis.

Protein–protein network analysis provided a set of genes/proteins consisting of the union of three MCODE clusters and a CytoHubba set of six highly connected genes: *APOA2*, *APOB*, *APOC2*, *APOC3*, *APOE*, *APOM*, *C4B*, *C4BPA*, *C9*, *FGG*, *GC*, *HP*, *HRG*, *LPA*, *SAA2-SAA4*, and *SERPIND1.*

The identified DEPs were primarily involved in GO categories related to the binding, clearance, and remodeling of lipoprotein particles. Additionally, GO terms demonstrated significant enrichment for complement activation, regulation of blood coagulation, and fibrinolysis.

An analysis of the KEGG and Reactome pathways identified significant enrichment in pathways associated with lipoproteins. The involvement of the identified DEPs in the innate immune system, complement, and coagulation cascades may serve as a potential reference point for future research aimed at elucidating the pathological mechanisms of the disease.

Based on the evidence to date, the mechanism of action of bisphosphonate-induced ONJ appears to involve disruption of osteoblast and osteoclast homeostasis, as well as alterations in coagulation, oxidative stress, and bone cell death. Ultimately, altered lipid metabolism is hypothesized to be linked to the pathophysiology of the disease [10].

A substantial amount of research has demonstrated a correlation between disrupted lipid metabolism and metabolic dysfunction in bones. However, the precise relationship between bone metabolism and lipid metabolism remains uncertain [21,22,23].

Recent studies have established a correlation between cholesterol metabolism and osteoblasts, a class of mesenchymal cells that regulate the formation and mineralization of bone matrix. By controlling the growth and activation of osteoblasts and osteoclasts, cholesterol and its metabolites impact bone homeostasis [24,25].

The process of lipid internalization and utilization by osteoblasts is facilitated by receptors and catabolic enzymes that are intrinsic to this cellular type. Disruption of this functionality can lead to skeletal impairments and simultaneous alterations in whole-body lipid homeostasis [26].

The metabolism of plasma lipids is influenced by apolipoprotein E (ApoE). The *APOE* gene variants exerted the most pronounced effect on plasma triglyceride levels. ApoE has been demonstrated to enhance lipoprotein lipase activity and is present on high-density lipoprotein particles that are rich in triglycerides. Recombinant ApoE binds to members of the LDL receptor family, as has recently been shown [27]. Moreover, it has been discovered that ApoE expression in macrophages controls microRNA-regulated NF-κB signaling, which in turn limits the production of inflammatory cytokines [28].

Furthermore, apolipoprotein B (ApoB) is implicated in a number of metabolic activities beyond cholesterol transport. Two significant inflammatory pathways, namely the mitogen-activated protein kinases and nuclear factor kappa-light-chain-enhancer of activated B cell pathways [10], have been identified as involving ApoB. Likewise, research has yielded evidence of a correlation between ApoB and bone mineral density (BMD) at various skeletal locations. However, the precise function of ApoB in bone metabolism remains to be elucidated [29].

Serum amyloid A (SAA) represents a family of acute-phase response proteins [30]. They are integral components of the innate immune system and may also function as circulating biomarkers of inflammation [31]. Previous studies have shown that SAAs are lipid-soluble proteins secreted into the circulation, almost all of which are bound to HDL, or very low-density lipoprotein, suggesting its involvement in lipoprotein metabolism [32]. The SAA family is associated with the metabolism of cholesterol. The binding of SAA to HDL can cause HDL to be transformed from a normally anti-inflammatory particle to a dysfunctional, pro-inflammatory particle [32]. To determine how SAA contributes to the balance between osteogenesis and adipogenesis in BMSCs, its effects on bone metabolism were investigated. It was established that SAA possesses the capacity to stimulate proliferation, inhibit osteogenesis, and facilitate adipogenesis. The suppression of osteogenic differentiation, coupled with augmented lipid production, contributed to a reduction in bone formation [33]. Furthermore, numerous studies have demonstrated that SAA interacts with complement system components to exert regulatory effects on inflammatory processes, thereby mediating the activation of immune cells [34]. For example, evidence indicates that SAA recruits C4-binding protein to impede the assembly of the terminal complement complex in an environment of normal serum [35].

Several genetic screening approaches have now been employed to examine a significant number of genes that may be genetically associated with the development of MRONJ (e.g., [16,36,37,38]). Although the results have been heterogeneous, gene experiments have revealed probable cellular pathways involved in the pathogenesis of MRONJ [39]. Studies have identified an association between the presence of single-nucleotide polymorphisms in *CYP2C8*, *PPARG*, and *VEGF* and an elevated risk of developing bisphosphonate-related osteonecrosis of the jaw (BRONJ) [39]. These genes are integral to both direct and indirect angiogenesis, as well as to lipid metabolism pathways. *CYP2C8* is responsible for the conversion of arachidonic acid to epoxyeicosatrienoic acid [40], which exerts significant effects on vascular tone and cardiovascular homeostasis [41]. The *PPARG* gene has been demonstrated to exert control over the expression of genes implicated in the transport and metabolism of lipids. Such genes include *FABP4*, *LXRA*, and *PGR*, all of which play important roles in regulating the expression of genes related to angiogenesis and vascular development [42].

*VEGF* and its active protein form, VEGFA, are required to regulate angiogenesis and vascular growth [43]. These findings indicate a correlation between the risk of BRONJ and the control of these mechanisms, notably angiogenesis and lipid metabolism [39].

Apart from its function as a complement inhibitor, studies have demonstrated that C4b-binding protein (*C4BP*) stimulates cell death during apoptosis. Moreover, bacteria and tumors can use this mechanism to evade complement-mediated lysis. Although initially recognized as a vital inhibitor of the complement cascade, growing evidence suggests that *C4BP* may also function independently of the complement system to support cell survival, protect the body from autoimmune damage, and alter the pathogenic potential of microorganisms [44]. Preliminary findings indicate that the inhibitory effects of circulating C4b-binding protein alpha chain (*C4BPA*) on complement activity and complement-induced platelet reactivity may contribute to the antiplatelet effects associated with this protein [45].

Notably, osteonecrosis of the femoral head has been shown to cause variable expression of proteins in the hip articular cartilage, including *C4BP* [46].

Scientific investigation has indicated that fibrinogen gamma chain (*FGG*) could also have a role in cartilage degradation, acting in a manner similar to that of femoral head osteonecrosis [47]. Furthermore, it has been demonstrated that individuals with MRONJ evidence altered *FGG* expression in their whole saliva [12]. As posited by the authors, a hypercoagulable milieu is inferred based on the observation that fibrinogen and lipoprotein levels are elevated at the site of bone lesion development [48,49,50]. The fact that high levels of lipoprotein and fibrinogen result in the activation of platelets and a delay in thrombolysis [51] adds more evidence to the theory that hypercoagulability and inadequate proteolytic degradation of fibrin are substantially linked with disease progression in patients with osteonecrosis.

Histidine-rich glycoprotein (*HRG*) ligands also include fibrinogen. Under various circumstances, such as when tissue damage occurs, the histidine-rich region of HRGP enhances ligand binding after interacting with Zn^2+^ or being exposed to low pH. Recent findings indicate that *HRG* also has the capacity to regulate the polarization of macrophages and to exert influence over a number of additional physiological processes, including angiogenesis, the immune response against tumors, fibrinolysis and coagulation, the removal of soluble immune system complexes, and the phagocytosis of necrotic/apoptotic cells [52].

An up-regulated protein detected in our experiments was the anti-coagulant heparin cofactor 2 (*SERPIND1*). By affecting the blood coagulation cascade, it inhibits thrombin in blood plasma, thereby preventing excessive blood clotting [53]. Playing an anticoagulant role, *SERPIND-1* elevation was observed in plasma during the healing of the radiation therapy-associated skin lesions [54]. Although it is assumed to participate in the process of responding to vascular injury, there remains a significant gap in our understanding regarding its physiological function [55]. *SERPIND1* also acts as a chemotactic agent for neutrophils and monocytes [56]. The release of chemotactic peptides is observed when *SERPIND1* is subjected to partial degradation by neutrophil proteases. This indicates a potential direct involvement of *SERPIND1* in inflammatory processes as a factor within the acute phase response signaling pathway [57]. Furthermore, *SERPIND1* may facilitate the wound healing process by acting as a modulator of thrombin-mediated mitogenic or chemotactic activities [58]. *SERPIND1* has been the subject of extensive study and is understood to exert an inhibitory influence in biological systems. It plays an essential role in vital physiological processes, including blood coagulation. Our experiments revealed an unexpected upregulation of *SERPIND1*. These findings may indicate that the activation of coagulation represents an initial response to bisphosphonate treatment, which is subsequently diminished during the reparative phase by the action of coagulation inhibitors. Given its anticoagulant properties, it can be posited that the rise in *SERPIND1* during this phase may serve as an indicator of the healing of drug-related necrotic changes.

It is acknowledged that the complement system is a key element of innate immunity. Recently, the complement system has been shown to have a larger role in the immune response and is now understood to serve as a conduit between innate and adaptive immunity [59]. The complement activation classical pathway results in the structure of the complement membrane attack complex (MAC), which is assembled by subsequent binding of molecules including as many as 16–18 C9 molecules. MAC, which causes osmolysis by forming pores in the plasma membrane of microbial pathogens or cellular targets, has been identified as a cytolytic agent of innate and adaptive immunity [60]. From this perspective, the identified up-regulation of complement component C9 in the saliva of MRONJ patients is an expected consequence of the role of MAC at the high prevalence observed in necrotic tissues [61].

Until recently, the prevailing view was that osteonecrosis was caused solely by the death of osteoblasts and osteocytes, as well as anomalous osteoclast activity. However, recent evidence indicates that immune system dysfunction may play a significant role in the onset of MRONJ [62]. The most recent research has postulated a correlation between atypical immune responses and immune cell infiltration in osteonecrotic tissues exhibiting signs of uncontrolled inflammation [63,64,65]. This raises the question of how distinct immune cells regulate inflammation in osteonecrosis. Recent studies point to the conclusion that in necrotic bone tissue, the continuous recruitment of innate (macrophages, neutrophils, and dendritic cells) and adaptive (T cells and B cells) immune cells by inflammatory cytokines and chemokines amplifies the inflammatory response, which in turn fosters bone resorption and inhibits bone formation. This process ultimately contributes to the development of osteonecrosis [66,67,68]. From a genetic perspective, the authors of study [66] evidenced a correlation between specific immune cell signatures, including CD20%-positive lymphocytes, CD62L-positive monocytes, and CD33br HLA DR+ CD14-cells, and an elevated risk of osteonecrosis. In contrast, EM CD4+ activated cells and DP (CD4+ CD8+) T cells were found to be associated with a reduced risk of osteonecrosis. It is noteworthy that a potential reduction in CD45 expression on immature myeloid-derived suppressor cells was observed to correlate with osteonecrosis [66].

Similarly, a patient with BRONJ was previously demonstrated to exhibit a high proportion of native CD4+ T cells and M0 macrophages, while resting mast cells, NK cells, and eosinophils were diminished [69]. Additionally, a positive correlation was observed between resting dendritic cells and gamma delta T cells. Furthermore, 36 immune genes that exhibited differential expression were investigated. GO enrichment analysis evidenced that peptidyl-tyrosine modification, myeloid leukocyte migration, leukocyte chemotaxis, and regulation of chemokine production were the most frequently differentially expressed immune-related genes. Furthermore, the KEGG analysis demonstrated that the differentially expressed genes (DEMGs) were predominantly associated with cytokine-cytokine receptor interaction, the IL-17 signaling pathway, and the NF-κB signaling pathway [69].

## 4. Material and Methods

### 4.1. Inclusion Criteria for Patients with MRONJ

Here, we conducted a case–control study recruiting 10 subjects (2 men, 8 women, age: 67.6 ± 7.2 years) who had a history of MRONJ and had received treatment at the Department of Dentistry and Maxillofacial Surgery of the Faculty of Medicine of the University of Pavol Jozef Šafárik in Košice, between the months of March and November 2023, and a ten-member control group (4 men, 6 women, age: 29.0 ± 9.8 years). All participants were enrolled with informed written consent. The study was conducted in accordance with the ethical standards outlined in the Declaration of Helsinki and received approval from the Ethics Committee of the Luis Pasteur University Hospital in Košice (118/EK/2022). All patients had been diagnosed with bisphosphonate-induced osteonecrosis of the mandible and maxilla. The diagnosis was made in accordance with the criteria established by the American Association of Oral and Maxillofacial Surgeons (AAOMS), namely: 1. The patient is currently undergoing or has undergone treatment with a medication that carries a high risk of adverse effects. 2. The bone area was exposed to the oral environment for a period exceeding eight weeks, despite the European task force on MRONJ’s recommendation that such a lengthy observation period for potential MRONJ manifestation is unnecessary [70]. 3. The patient has not undergone radiotherapy to the head and neck region, and there is no evidence of metastasis of cancer in the jaw and temporal region [71]. The study was limited to participants with necrotic bone exposed to the oral cavity, corresponding to stages 1 through 3, as defined by the classification system.

**Figure 10:**
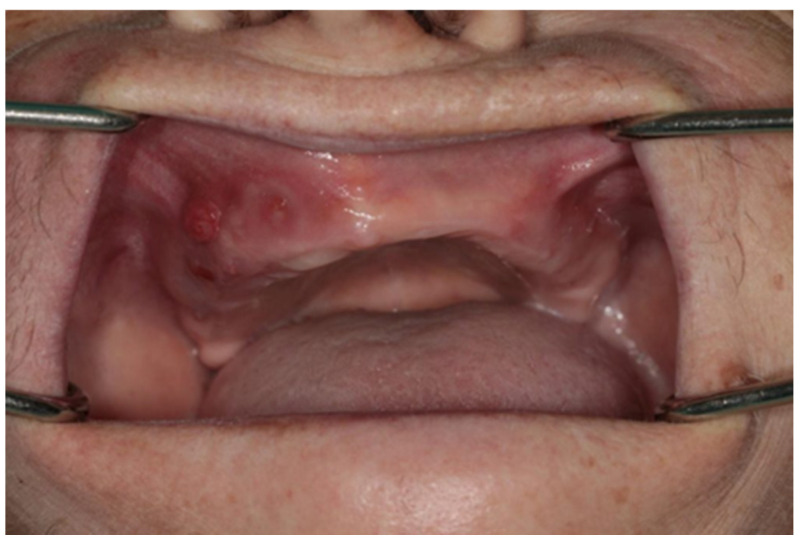
A 68-year-old female patient (no. 10) with postmenopausal osteoporosis who had been on antiresorptive treatment with zoledronic acid (Aclasta) administered intravenously once a year for three years presented with bisphosphonate-induced osteonecrosis.

**Figure 11:**
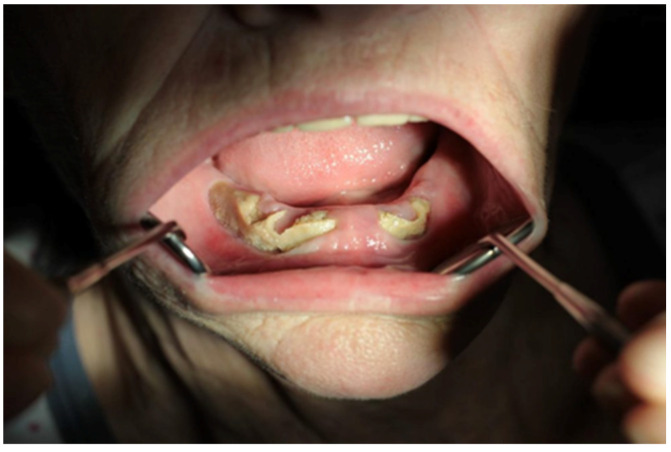
Bisphosphonate-induced osteonecrosis in a 65-year-old female patient (no. 8) with metastatic breast cancer. The patient was treated for 2 and a half years with zoledronic acid (Osporil) administered intravenously once a month, along with the cytostatic therapy Capecitabine.

In the experimental group, osteonecrosis was observed more frequently in the *mandible* (7 patients) than in the maxilla (3 patients). It occurred predominantly in patients primarily treated for cancer, whereas 1 patient was treated for osteoporosis without a history of cancer (Table 2). Table 3 presents the basic data on the type and duration of treatment for the patients. In the oral cavity of the patients, there were mucosal defects of various sizes, including protrusion of persistent fistulae (Figure 10) and exposed alveolar bone (Figure 11). 

### 4.2. Sample Processing

#### 4.2.1. Saliva Sampling

To circumvent the potential impact of diurnal variation in saliva composition, saliva samples were collected between 9:00 a.m. and 11:00 a.m. Prior to collection, participants were requested not to perform oral hygiene routines or consume food in the morning and to rinse their mouths with 50 mL of water. Unstimulated, whole saliva collections were performed by expectorating into a 50 mL sterile tube placed on ice. To prevent the enzymatic activity of proteases, a volume of 1 µL of protease inhibitor cocktail (Protease Inhibitor Cocktail, Merck Life Science, Darmstadt, Germany) was combined with 1 mL of saliva. Collection times averaged 5 to 10 min, depending on the salivation of the patients. The samples were subjected to centrifugation (12,000× *g*, 30 min, 4 °C) to accomplish the removal of bacteria, dead epithelial cells, and cellular debris. The supernatants were subsequently frozen and stored at −80 °C.

#### 4.2.2. Total Protein Concentration Assay

The protein content in saliva samples was measured using the Bradford assay (Quick Start Bradford Protein Assay, Bio-Rad, Hercules, CA, USA) with standards of bovine serum albumin. A UV-VIS spectrophotometer (UV-3600 Spectrophotometer, Shimadzu Corp., Kyoto, Japan) was used to measure the absorbance at 595 nm.

#### 4.2.3. Filter-Aided Sample Preparation

The sample for LC-MS/MS was processed employing the FASP (Filter-Aided Sample Preparation) method. A volume of 100 µg of proteins from each saliva sample was transferred to a centrifuge tube with a 10 kDa MWCO filter (Microcon Ultra−0.5 mL, Merck Millipore, Billerica, MA, USA). The sample was subjected to centrifugation at 14,000× *g* for a period of 10 min at a temperature of 20 °C, with the objective of removing the supernatant. The filter was then exposed to centrifugation under the conditions previously described, utilizing 200 µL of urea with an 8 mol L^−1^ in a 25 mmol L^−1^ ammonium bicarbonate (AB). Disulfide bonds of proteins were reduced with 200 µL of 50 mmol L^−1^ DTT in 8 mol L^−1^ urea and 25 mmol L^−1^ AB. The sample was maintained at 37 °C for a period of 60 min and then processed through a second centrifugation at 14,000× *g* for 15 min at 20 °C. The addition of 100 µL of a solution containing iodoacetamide (IAA) at a concentration of 50 mmol L^−1^ in 8 mmol L^−1^ urea and 25 mmol L^−1^ AB resulted in the alkylation of the cysteine. This was followed by incubation in the dark for 45 min at 37 °C and centrifugation at 14,000× *g* for 10 min at 20 °C. Finally, the sample was washed two consecutive times with 200 µL AB and centrifuged under the same conditions as the last time. To initiate the enzymatic digestion of proteins, trypsin was added to an AB solution with a concentration of 12.5 mmol L^−1^ and a trypsin/protein ratio of 1:30 (*w*/*w*). The sample was then incubated without agitation overnight at 37 °C. The peptides were transferred to a collection tube using centrifugation at 14000× *g* for 15 min at 20 °C. Following this, the centrifugation filter membrane was washed on two occasions with 200 µL of deionized water under the same centrifugation conditions that were employed during the preceding steps. The sample was subjected to vacuum drying in a centrifugation concentrator (Labconco CentriVap Acid-Resistant Vacuum Concentration System, Labconco, Kansas City, MO, USA) at 55 °C and then frozen at −80 °C. For the purpose of MS analysis, the peptides were reconstituted in a solvent solution comprising 50 µL of 3% ACN (*v*/*v*) with 0.1% FA (*v*/*v*) and homogenized for a period of one minute using ultrasound.

### 4.3. Mass Spectrometry

The processed samples were measured using a sensitive high-resolution mass spectrometer, the Thermo Scientific™ Orbitrap Eclipse™ Tribrid™ (Thermo Scientific, Waltham, MA, USA), coupled with a nano-liquid chromatograph, the Thermo Scientific™ Ultimate 3000. As the ion source, Thermo Scientific™ EASY-Spray™ NSI source was used.

A volume of 1 μL of the samples was injected at a flow rate of 15 μL min^−1^ onto a 5 mm long chromatographic trap column (PepMapTM Neo Trap Cartridge, Thermo Scientific, Waltham, MA, USA) with a C18 particle size of 5 μm. The mobile phase used during the injection was water with 0.1% FA. After being captured on the column, the peptides were eluted onto an analytical separation column (Thermo Scientific™ EASY-Spray™) with a length of 50 cm and a C18 particle size of 2 μm using a pre-set time-dependent gradient. The column was heated to 40 °C during the measurement. In order to achieve the necessary separation, two different mobile phases were employed throughout the gradient: phase A, consisting of 98% H_2_O, 2% ACN, and 0.1% FA, and phase B, consisting of 80% ACN, 20% H_2_O, and 0.1% FA. The elution gradient, with a total duration of 140 min, proceeded as follows: the start of the gradient consisted of a mixture of 98% mobile phase A and 2% mobile phase B; over the next 100 min, the composition changed linearly to 24% mobile phase B; after 20 min of linear increase, mobile phase B reached 40%, and after another 10 min, it reached 90%. The column was washed with 90% mobile phase B for 10 min, and the remaining time was used for equilibration of the analytical column to the initial gradient composition of 2% mobile phase B.

Following the separation of peptides on the analytical column, ionization occurred at the end of the EASY-Spray™ column emitter (Thermo Scientific, Waltham, MA, USA) under a voltage of 2000 V, whereby the peptides were sprayed into the mass spectrometer with the following set parameters: positive ion mode, with full MS1 scans on the Orbitrap analyzer at a resolution of 120,000 and a maximum injection time of 100 ms. HCD fragmentation with energy set to 30% was followed by MS2 scans with resolution set to 30 000 and a maximum injection time of 50 ms. Preset precursor selection parameters: charge state range of 2–5 and an intensity threshold of 2 × 10^4^, dynamic exclusion time 60 s, and mass tolerance 10 ppm. Full MS1 scans were repeated every 2 s, and the number of MS2 scans between two full MS1 master scans varied automatically depending on the number of suitable precursors detected in the previous MS1 scan.

The measured RAW files were analyzed using the proteomics software Proteome Discoverer 2.5 (Thermo Scientific™), which is designed for the processing and evaluation of proteomic data. The identification of the detected proteins was achieved by utilizing a combination of three integrated search engines: MS Amanda 2.0, Sequest HT, and Mascot. For MS Amanda 2.0 and Sequest HT, the UniProt database for Homo sapiens (version 2023_02) containing 182,026 protein sequences was used as the search database. For Mascot, the SwissProt Human database was employed. Trypsin was chosen as the proteolytic enzyme, and up to two missed cleavage sites were allowed. The MS1 tolerance was assigned a value of 10 ppm, while the MS2 tolerance was assigned a value of 0.02 Da.

For quantification, we included both unique and razor peptides, relying on precursor abundance determined by intensity. The data were normalized using the total peptide amount, providing a robust baseline for comparison. Protein ratios were calculated using a pairwise ratio-based method, ensuring accurate relative quantification, and statistical significance was assessed with a *t*-test to validate the findings.

### 4.4. Bioinformatics Analysis

#### 4.4.1. Gene Ontology and Pathway Analysis

To verify and refine the list of significant DEPs, it was cross-referenced with the GeneCards human gene database (https://www.genecards.org, (accessed on 14 November 2024)). A framework for open-source visualization and integration of molecular interaction networks and biological processes was made available via the Cytoscape 3.10.2 [72] software. The Cytoscape BiNGO 3.0.5 [73] extension was employed to examine the DEPs with respect to overrepresentation in the hierarchical gene ontology (GO). The cellular components (CC), molecular functions (MF), and biological processes (BP) of genes were used to create hierarchical categories in a GO analysis to clarify genetic regulatory networks of interest. These categories allowed for the comparison and analysis of genes across control and MRONJ subjects. Benjamini and Hochberg multiple testing with a cut-off of *p* < 0.05 was used for enrichment analysis of BP, MF, and CC terms. The ReactomeFI pathway database plug-in version 8.0.6 [74] implemented in Cytoscape was chosen for the pathway enrichment analysis of the DEPs. The statistical significance of the findings was ascertained by applying a significance threshold of *p* < 0.05 to identify pathways that were statistically enriched. Furthermore, a joint Kyoto Encyclopedia of Genes and Genomes (KEGG) and Reactome pathway analysis was conducted in Cytoscape using ClueGo 2.5.10 [75].

#### 4.4.2. Protein–Protein Interaction Network Analysis

The network of differentially expressed genes/proteins was constructed with the help of the STRING database, which allows for the discovery and prediction concerning protein–protein interactions (PPIs). For PPI visualization in Cytoscape, StringApp 2.1.1 [76] was selected. A credibility score greater than 0.4 was applied as the cut-off point. The Molecular Complex Detection (MCODE) 2.0.3 [77] algorithm was used to cluster a given network to find highly interconnected domains and identify the most significant modules. The following configuration parameters were chosen as the selection criteria for significant modules: Maximum Depth from Seed: 100; Degree Cutoff: 2, Node Score Cutoff: 0.05, K-Core: 2.

Using the CytoHubba 0.1 plug-in [78], the hub genes were retrieved to achieve balance between the core genes and prevent missing the crucial genes. CytoHubba algorithms were employed to recognize hub nodes or subnetworks contained within a particular network, including the Degree (Deg), Density of Maximum Neighborhood Component (DMNC), Maximal Clique Centrality (MCC), and Maximum Neighborhood Component (MNC) algorithms. In addition to the local-based methods, global-based methods were employed, including Betweenness (BC), BottleNeck (BN), Closeness (Clo), EcCentricity (EC), Edge Percolated Component (EPC), Radiality (Rad), and Stress (Str) algorithms [78].

To identify genes common to all computational algorithms, the UpSet plot was used. A volcano plot was constructed using a 1.5-fold cut-off value and a statistical threshold of *adj. p* < 0.05. Volcano, bubble, and UpSet plots were generated utilizing the https://www.bioinformatics.com.cn (accessed on 23 January 2024) platform [79].

## 5. Conclusions

The objective of the study was to identify specific salivary proteins/genes underlying bisphosphonate-induced osteonecrosis of the jaw and to investigate their function in the mechanisms of MRONJ pathogenesis.

Although saliva possesses a multitude of beneficial characteristics, it is not without certain limitations, which in turn restrict the scope of salivary proteomics. As a reflection of both oral and systemic health, saliva can be viewed as a diagnostic indicator. However, the levels of specific biomolecules may not always align with those observed in serum, introducing a degree of inconsistency.

It should also be acknowledged that the sample sizes in the study in both the patient and control groups were relatively small, which may limit the reliability of the findings. To achieve a more in-depth comprehension of the potential interconnections between these proteins and MRONJ, it would be beneficial to undertake additional large-scale studies. In the ongoing pursuit of universal biomarkers for disease, it would be advantageous to consider the inclusion of a larger patient cohort, comprising individuals with osteoporosis. At the same time, however, it was challenging to identify individuals who did not have an associated condition because of the low prevalence of MRONJ patients during the specified time frame.

Another point of contention is the age disparity between individuals in the MRONJ and control groups. The lower mean age of the control group was selected due to the difficulty in identifying healthy individuals without systemic or other diseases who are aged 60 years and older.

In conclusion, despite the limitations, the proteomic study demonstrated that MRONJ has links to signaling pathways associated with lipoproteins. Additionally, the research indicated changes in saliva-affecting proteins related to pathways such as the innate immune system, complement, and coagulation cascades. However, further studies are necessary to validate these preliminary findings and select an optimal biological marker for the diagnosis of MRONJ.

## Figures and Tables

**Table 1:** The list of the top ten significantly down-regulated and significantly up-regulated differentially expressed proteins in MRONJ according to the fold change.

Gene	Protein Description	Fold Change	*p*-Value
Up-regulated			
*APOC3*	Apolipoprotein C-III	2.96144	7.97 × 10^−5^
*FGG*	Fibrinogen gamma chain. isoform CRA_a	3.14454	6.70 × 10^−6^
*HBA2*	Alpha-globin chain	3.15801	0.000754
*IGHV1-2*	IGHV1-2 protein	3.24092	0.007659
*HBA2*	Mutant hemoglobin subunit alpha 2	3.24610	0.001417
*HBB*	Beta-globin (fragment)	3.37295	0.000802
*F5-20*	F5-20	3.40177	0.00041
*HRG*	Histidine-rich glycoprotein	3.65924	0.000192
*HBB*	Hemoglobin beta chain	3.67987	0.000186
VHCH1	Immunoblobulin G1 Fab heavy chain variable region	3.96384	6.65 × 10^−6^
Down-regulated			
*GSTT2B*	Glutathione S-transferase theta-2B	−2.50635	0.040709
*IgH*	Ig heavy chain variable region	−2.55639	0.000329
*SCGB2A1*	Mammaglobin-B	−2.72738	0.003005
*SCGB1D1*	Secretoglobin family 1D member 1	−2.80591	0.001932
*LAMTOR5*	Ragulator complex protein LAMTOR5	−2.82623	0.010758
*IGLC7*	Immunoglobulin lambda constant 7	−2.93236	0.000184
*GMDS*	GDP-mannose 4.6 dehydratase	−3.01159	0.018747
*U7*	U7	−3.07097	0.001064
*HP*	Haptoglobin	−3.62593	1.96 × 10^−6^
*ANXA4*	Annexin A4	−4.10780	2.23 × 10^−9^

**Table 2:** Overview of patients’ primary diagnoses.

Sex	Men	Women
Oncological diagnosis	metastatic prostate cancer/2	metastatic breast cancer/6 multiple myeloma/1
Non-oncological diagnoses		osteoporosis/1
Count	2	8

**Table 3:** Data on the type and duration of the treatment received by patients.

Patient N	Diagnosis	Medication	Treatment/Weeks Duration (Months)	Administration Method
1	metastatic prostate cancer	zoledronic acid (Zometa)	24	intravenously
2	metastatic prostate cancer	ibandronic acid (Ossica)	12	intravenously
3	breast cancer	zoledronic acid (Aclasta)	60	intravenously
4	breast cancer	pamidronic acid (Pamifos)	72	intravenously
5	breast cancer	ibandronic acid (STADA)	17	intravenously
6	breast cancer	pamidronic acid (Pamifos)	54	intravenously
7	breast cancer	zoledronic acid (Sandoz)	42	intravenously
8	breast cancer	zoledronic acid (Osporil)	30	intravenously
9	multiple myeloma	zoledronic acid (Zomikos)	10	intravenously
10	osteoporosis	zoledronic acid (Aclasta)	36	intravenously

## Data Availability

Data are contained within the article or Supplementary Materials.

## Supplementary Materials

The following supporting information can be downloaded at: https://www.mdpi.com/article/10.3390/ijms252212405/s1.
