# Supplementary figures and images for: Insights into Medication-Induced Osteonecrosis of the Jaw Through the Application of Salivary Proteomics and Bioinformatics
Source: Int J Mol Sci. 2024 Nov 19;25(22):12405. doi: 10.3390/ijms252212405 (PMC11594355; doi:10.3390/ijms252212405)

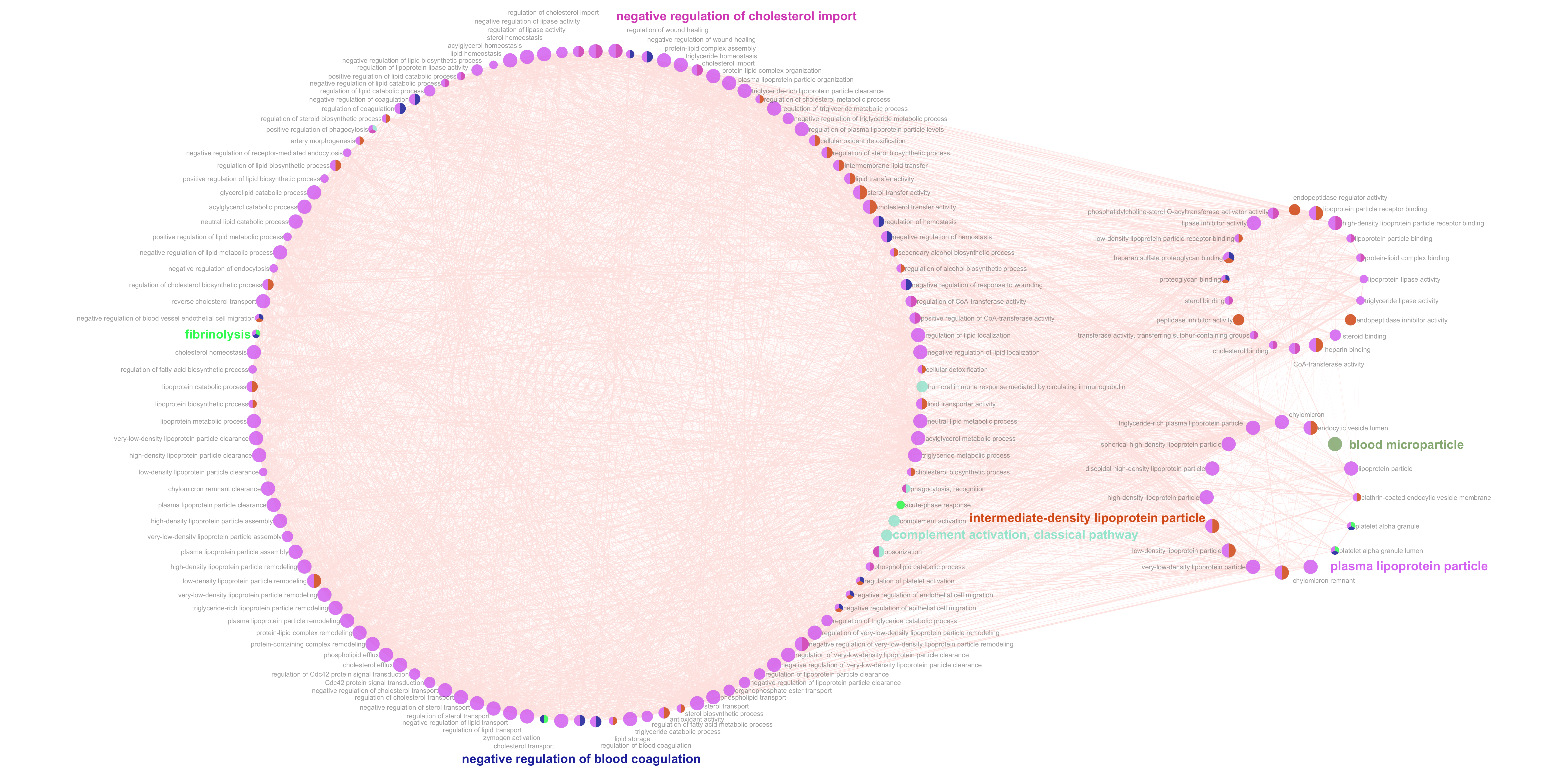

Supplement: Supplementary file 1 [file ijms-25-12405-s001.zip › Figure S1.png]
